# Thermokinetic profile of NDM-1 and its inhibition by small carboxylic acids

**DOI:** 10.1042/BSR20180244

**Published:** 2018-04-13

**Authors:** Qian Wang, Yuan He, Rui Lu, Wen-Ming Wang, Ke-Wu Yang, Hai Ming Fan, Yi Jin, G. Michael Blackburn

**Affiliations:** 1 Key Laboratory of Synthetic and Natural Functional Molecule Chemistry of Ministry of Education, College of Chemistry and Materials Science, Northwest University, 1 Xue Fu Avenue, Xi’an 710127, P.R. China; 2 School of Chemistry, Cardiff University, Cardiff CF10 3AT, U.K.; 3 Department of Molecular Biology and Biotechnology, University of Sheffield, Sheffield S10 2TN, U.K.

**Keywords:** carbapenemase, enzyme activity, isothermal titration calorimetry, metalloenzymes, molecular mechanisms, small molecules

## Abstract

The New Delhi metallo-β-lactamase (NDM-1) is an important clinical target for antimicrobial research, but there are insufficient clinically useful inhibitors and the details of NDM-1 enzyme catalysis remain unclear. The aim of this work is to provide a thermodynamic profile of NDM-1 catalysed hydrolysis of β-lactams using an isothermal titration calorimetry (ITC) approach and to apply this new method to the identification of new low-molecular-weight dicarboxylic acid inhibitors. The results reveal that hydrolysis of penicillin G and imipenem by NDM-1 share the same thermodynamic features with a significant intrinsic enthalpy change and the release of one proton into solution, while NDM-1 hydrolysis of cefazolin exhibits a different mechanism with a smaller enthalpy change and the release of two protons. The inhibitory constants of four carboxylic acids are found to be in the micromolar range. The compounds pyridine-2,6-dicarboxylic acid and thiazolidine-2,4-dicarboxylic acid show the best inhibitory potency and are confirmed to inhibit NDM-1 using a clinical strain of *Escherichia coli*. The pyridine compound is further shown to restore the susceptibility of this *E. coli* strain to imipenem, at an inhibitor concentration of 400 μM, while the thiazoline compound also shows a synergistic effect with imipenem. These results provide valuable information to enrich current understanding on the catalytic mechanism of NDM-1 and to aid the future optimisation of β-lactamase inhibitors based on these scaffolds to tackle the problem of antibiotic resistance.

## Introduction

**Figure 1:**
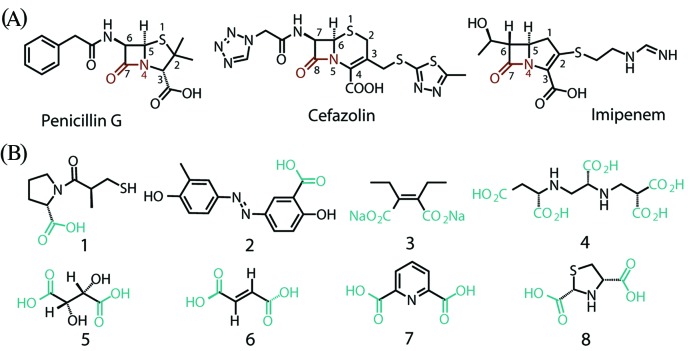
Structures of representative β-lactam antibiotics and carboxylic acid inhibitors D-Captopril **1**, salicylic acid derivative **2**, ME1071 **3**, AMA **4**, D-(−)-tartaric acid **5**, fumaric acid **6**, pyridine-2,6-dicarboxylic acid **7** and *rac*-thiazolidine-2,4-dicarboxylic acid **8**.

β-Lactam antibiotics (Figure 1A), including penicillins (e.g. penicillin G), cephalosporins (e.g. cefazolin), monobactams and carbapenems (e.g. imipenem), are the most widely used agents for treating clinical bacterial infections [1]. Among these, carbapenems, featuring a five-membered ring fused to the β-lactam core structure, are an important class of β-lactam antibiotics widely used in the treatment of serious bacterial infections caused by bacteria expressing extended-spectrum β-lactamases (ESBLs) [2,3]. However, the overuse of this class of antibiotic has led to decreased bacterial susceptibility towards these compounds, mostly via mechanisms such as antibiotic inactivation by carbapenemases, reduced bacterial membrane permeability, altered penicillin binding proteins (PBPs) and/or efflux of antibiotics [4–8]. Of these mechanisms, production of carbapenemases poses the most serious threat, not only because carbapenemase-producing bacteria tend to have higher level of resistance to carbapenems, but also because the carbapenemase gene in mobile genetic elements is easy to transfer across species, giving rise to nosocomial outbreaks of carbapenem-resistant pathogens [9]. According to amino acid homologies, carbapenemases have been classified into A, B and D groups in the Ambler system. Class A and D enzymes contain a catalytic serine residue in the active site to aid the hydrolysis of the C–N bond through formation of a covalent acyl-enzyme adduct, whereas class B enzymes (metallo-β-lactamases, MβLs) require the presence of zinc to activate water for nucleophilic attack on the substrate [10].

Currently, several inhibitors of serine-β-lactamases, including clavulanic acid, sulbactam and tazobactam have been clinically approved to work in synergy with β-lactams, while others (e.g. avibactam and MK-7655) are in clinical trial. While such combination therapy is useful in tackling infections due to serine-β-lactamases, there are currently no inhibitors approved for use against bacteria harbouring MβLs [11–14]. Of particular clinical importance, New Delhi metallo-β-lactamase-1 (NDM-1), first discovered in 2008 in a strain of *Klebsiella pneumoniae* isolated in India, is now one of the most abundant MβLs in clinic [15,16]. It has been found in species of *Enterobacteriaceae, Acinetobacter baumannii, Vibrio cholerae, Stenotrophomonas* and *Pseudomonas aeruginosa*, and accounts for 60% of autochthonous human infection isolates according to a survey of data from 2009 to 2012 [16,17]. Hence, a mechanistic study [18–20] of NDM-1 and the design of potent NDM-1 inhibitors for combination therapy are active areas for antimicrobial research [21].

Among the reported NDM-1 inhibitors, carboxylic acid-containing compounds are one of the most prominent categories [22,23]. D-Captopril **1** (Figure 1B) has been shown to be a potent competitive inhibitor of NDM-1 with *K*_i_ 1.3 μM [24,25], while a salicylic acid derivative **2** was predicted to inhibit NDM-1 in a molecular docking study [26]. A maleic acid substitute ME1071 **3** was reported to inhibit NDM-1 with a *K*_i_ of 24 μM [27]. King et al. identified a natural product AMA **4** as a potent inactivator of NDM-1 with a *K*_i_ value of 11 nM and an IC_50_ of 4.0 μM [28,29]. Hence, it is important to investigate whether even simpler carboxylic acid derivatives may have good inhibitory potency for further development of clinical NDM-1 inhibitors.

Isothermal titration calorimetry (ITC) is a powerful technique capable of measuring real-time heat change rates in enzyme-catalysed reactions, a parameter directly proportional to the reaction rate which has thus become widely used for enzyme thermodynamic and kinetic studies [30–35]. However, this technique has not received much attention in work on MβLs. We have recently developed an ITC assay to study the thermokinetics of a B3 subclass MβL L1 and our results demonstrate that the calorimetric approach is a simple and efficient method in evaluating the kinetics of β-lactam hydrolysis. It has the additional advantages of being able to provide valuable information on the thermodynamic nature of enzyme catalysis, being compatible with optically turbid bacterial suspensions in contrast with conventional UV spectroscopy [36]. In this work, we sought to apply this calorimetric approach to a mechanistic study of a clinically relevant NDM-1 in the B1 subclass of MβLs, using three representative β-lactams: penicillin G, cefazolin and imipenem. In the second part of this work, the inhibitory potencies of four low-molecular-weight dicarboxylic acid compounds on NDM-1 were investigated. Of the compounds studied, D-(−)-tartaric acid **5** and fumaric acid **6** are two natural dicarboxylates with structural resemblance to **3** and **4**. Pyridine-2,6-dicarboxylic acid **7** was reported to be an effective inhibitor of IMP-1 and CphA [37]. Recently, derivatives of **7** were demonstrated to be highly selective inhibitors for B1 MβLs [38]. Thiazolidine-2,4-dicarboxylic acid **8** was shown to have a powerful effect on CcrA, Imis and L1 activities [39,40]. It is generally believed that carboxylate compounds exert the inhibitory effect via interaction with active site zinc atoms and current data have revealed a significant degree of selectivity of these compounds between different MβLs [39]. Hence, an evaluation of carboxylates **5–8** on NDM-1 provides valuable information for the exploration of promising scaffolds and the development of broad-spectrum inhibitors of MβLs. Given that NDM-1 remains a global challenge in medicinal chemistry, characterisation of this clinically important enzyme using a calorimetric approach will give strong support to ongoing studies on the mechanism and inhibition of NDM-1.

## Materials and methods

### Enzyme and reagents

Antibiotics (>98% purity) were purchased from Sigma–Aldrich Trading Co. Ltd (Shanghai, China). Compounds **1** (98% purity) and **5–7** were from the Aladdin Industrial Corporation (Shanghai, China). Compound **8** was from J&K Scientific Ltd (Beijing, China). All other chemicals were analytical grade. The expression plasmid pET26b–NDM-1 [41] for the production of recombinant NDM-1 enzyme was a kind gift from Professor Michael Crowder at Miami University, Florida, USA. *Escherichia coli* ATCC25922 was purchased from the American Type Culture Collection (ATCC). The clinical *E. coli* strain used was isolated from a blood culture of one individual patient in the First Affiliated Hospital of Xi’an Jiaotong University (Xi’an, China), and had previously been confirmed by DNA sequence to produce NDM-1 (unpublished data).

### NDM-1 gene expression and protein purification

Recombinant NDM-1 was produced and purified as previously described [41]. Briefly, recombinant *E. coli* cells containing the NDM-1 gene in pET26b plasmid were grown in Lysogeny Broth (LB) medium supplemented with 25 μg/ml kanamycin at 37°C and gene expression was induced by adding 1 mM IPTG and 50 μM ZnCl_2_. The culture was incubated overnight at 20°C before cells were harvested and then lysed by sonication. Protein was first loaded on a Q-Sepharose ion exchange column and eluted with a gradient of 0–500 mM NaCl in 30 mM Tris–HCl, pH 8.0. A Superdex 75 size exclusion column was used to further purify the target protein in the buffer of 30 mM Tris–HCl, pH 7.5 and 200 mM NaCl. The purity of fractions containing NDM-1 protein was checked using SDS–PAGE, and the concentration of purified protein was determined using UV-spectroscopy with extinction coefficient 27960 M^−1^ cm ^−1^ at 280 nm.

### UV-spectroscopic assay

IC_50_ values of compounds **1** and **5–8** were determined using a spectroscopic method [42]. Assays were carried out using an Agilent UV 8453 spectroscopy at 25°C, with 60 μM penicillin G as a substrate in a total volume of 1 ml buffer (50 mM Tris–HCl, pH 7.0, 100 mM NaCl). Reactions were initiated by addition of NDM-1 enzyme to a final concentration of 20 nM and changes in absorbance of penicillin G at 205 nm were recorded continuously for 30 s. Rates were also determined in the presence of inhibitor by pre-incubation with the enzyme for 30 min at RT before starting kinetic experiments. IC_50_ values were determined using GraphPad Prism5 software (GraphPadSoftware, La Jolla, CA, USA) by plotting percentage of inhibition against inhibitor concentration; average values from three measurements are reported.

### Calorimetric assays

Enzyme kinetics of NDM-1 and inhibition studies by carboxylic acid compounds were conducted using an ITC-200 calorimeter (Malvern Instruments Ltd., UK), with a reference cell loaded with deionised water and experiments carried out at 25°C with a stirring speed of 750 rpm.

To obtain the apparent enthalpy change (Δ*H*_app_) of enzyme-catalysed hydrolysis of antibiotics (penicillin G, cefazolin or imipenem), experiments were performed by injecting 20 μl of 1 mM antibiotic solution, preloaded in the syringe, into a sample cell filled with 210 μl of 50 nM purified NDM-1 in buffer of 50 mM Tris–HCl, pH 7.0, 100 mM NaCl. Real-time changes of heat-flow were recorded continuously until return to baseline. Control experiments were performed by titrating substrate to buffer and using those data in corrections for heat of dilution before values of Δ*H*_app_ were determined using the embedded Origin software, by dividing integrated heat by the molar quantity of substrate converted according to (eqn 1), where d*Q*/d*t* is the rate of heat production, *V* is the volume of the sample cell and [*S*]_*t* = 0_ is the substrate concentration:

(1) $$\Delta H=\frac{f_{t=0}^{\infty }\frac{\text{dQ}}{\text{dt}}dt}{\left(\text{V}{\left[\text{S}\right]}_{\left(t=0\right)}\right)}$$

Experiments were also repeated in HEPES and phosphate buffers and the intrinsic enthalpy change (Δ*H*_int_) of the reactions calculated according to (eqn 2) by plotting the measured Δ*H*_app_ as a function of Δ*H*_ion_, where Δ*H*_ion_ is the ionisation enthalpy of the buffer used and *n* represents the number of exchanging protons. The values of Δ*H*_ion_ used in the calculation were Δ*H*_ion_ (Tris–HCl) = 11.34 kcal/mol, Δ*H*_ion_ (HEPES) = 4.88 kcal/mol and Δ*H*_ion_ (phosphate) = 0.86 kcal/mol [43].

(2) $$\Delta H_{\text{app}}=\Delta H_{\mathrm{int}}+n\;\Delta H_{\text{ion}}$$

For steady-state kinetics of NDM-1, experiments were conducted in multi-injection mode, with 10 nM NDM-1 in the sample cell and 10 mM antibiotic (penicillin G, cefazolin or imipenem) in the syringe, both in buffer of 50 mM Tris–HCl, pH 7.0, 100 mM NaCl. Successive injections of antibiotic into the sample cell were made at 120 s intervals. Following (eqn 3), the change in thermal power (d*Q*/d*t*) from each titration was converted into the reaction rate (*υ*). These rates were used to fit to the Michaelis–Menten equation (eqn 4) to obtain the kinetic parameters *K*_M_ (Michaelis–Menten constant) and *k*_cat_ (turnover rates), where [*E*]_tot_ is the total concentration of active enzyme.

(3) $$\upsilon =\frac{\text{dP}}{\text{dt}}=\frac{1}{\text{V}\Delta H_{\text{app}}}\cdot \frac{\text{dQ}}{\text{dt}}$$

(4) $$\upsilon =\frac{k_{\text{cat}}{\left[\text{E}\right]}_{\text{tot}}[\text{S}]}{K_{\text{M}}+[\text{S}]}$$

Inhibition studies for NDM-1 with compounds **1** and **5–8** using calorimetric assay were performed by pre-incubating NDM-1 with the corresponding compound for 30 min prior to starting a multi-injection experiment with penicillin G, as described above. The concentrations of substrate and NDM-1 were the same as for the kinetic assay. Concentrations used for **1** were 2 and 20 μM, for **5** were 1 and 2 mM, for **6** were 100 μM and 1.5 mM, for **7** were 1 and 5 μM and for **8** were 2 and 10 μM. Competitive (*K*_ic_) and uncompetitive (*K*_iu_) inhibition constant values for each inhibitor were obtained by fitting calculated reaction rates (*υ*) at different inhibitor concentrations to (eqn 5) using nonlinear regression analysis in Origin software (embedded with ITC), with *K*_ic_ and *K*_iu_ set as shared parameters between all datasets, inhibitor concentration [*I*], [*E*]_tot_, [*S*] as independent variables and *υ* as dependent variable.

(5) $$\upsilon =\frac{k_{\text{cat}}{\left[E\right]}_{\text{tot}}[\text{S}]}{[\text{S}]\left(1+\frac{\left[\text{I}\right]}{K_{\text{iu}}}\right)+{\text{K}}_{\text{m}}\left(1+\frac{\left[\text{I}\right]}{K_{\text{ic}}}\right)}$$

Cell-based calorimetric assays were performed using a clinical *E. coli* strain expressing NDM-1 gene (*E. coli–*NDM-1) and a reference bacterial strain ATCC 25922 (β-lactamase negative). Experiments were performed as above, except that bacterial suspensions were added to the sample cell of the calorimeter at a concentration of 1 OD in the absence and presence of 500 μM **1, 7** and **8**, prepared in 50 mM Tris–HCl, pH 7.0, 100 mM NaCl buffer supplement with 0.1% Triton. The raw calorimetric signal was obtained after all penicillin G (20 μl of 1 mM) was injected.

### MIC determinations

The efficacies of compounds **1, 7** and **8** to work in synergy with an antibiotic were investigated by determining the minimum inhibitory concentrations (MICs) of imipenem towards the above clinical *E. coli*–NDM-1 strain using a broth microdilution method according to guidelines of the Clinical and Laboratory Standards Institute (CLSI) [44]. Single colonies of *E. coli*–NDM-1 were used to inoculate Mueller–Hinton medium and were grown at 37°C overnight. A volume of 100 μl of diluted bacteria culture at a density of 10^6^ colony forming units (CFU) per ml was added to a series of 2-fold diluted imipenem solutions prepared in 100 μl Mueller–Hinton medium, in the presence of compounds **1, 7** and **8** at 0, 200 and 400 μM. Results were obtained after incubating the microtitre plate at 37°C for a further 18 h and turbidity of bacterial growth was recorded using a plate reader.

## Results and discussion

**Figure 2:**
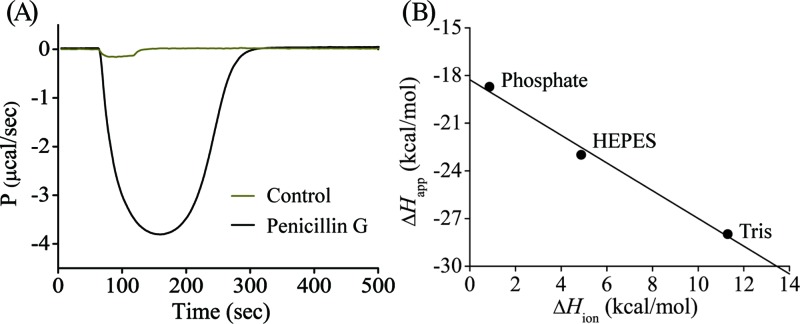
Calorimetric thermogram for the titration of penicillin G with NDM-1 in a single injection ITC assay and the determination of the intrinsic enthalpy of reaction (Δ*H*_int_) (**A**) Raw thermal power measured by titrating 20 μl of 1 mM penicillin G into 60 nM NDM-1 protein or buffer (control) at 25°C. (**B**) Determination of the intrinsic enthalpy change (Δ*H*_int_) of penicillin G catalysed by NDM-1 with linear regression of the apparent enthalpy change (Δ*H*_app_) for penicillin G over the protonation enthalpy of each buffer (Δ*H*_ion_) to give the intercept for Δ*H*_int_ and the slope for the number of protons (*n*) exchanged during catalysis.

**Figure 3:**
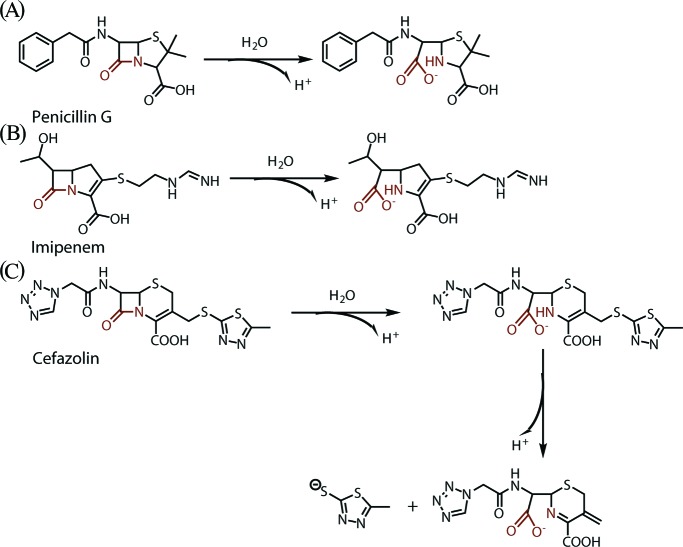
Schematic illustration of the proposed catalytic mechanism of NDM-1 towards three β-lactam antibiotics, highlighting the difference in number of protons released.

A single injection calorimetric experiment was performed to record the real-time heat change associated with NDM-1 catalysed hydrolysis of β-lactams and a raw calorimetric trace of titrating 20 μl of 1 mM penicillin G into 210 μl of 50 nM NDM-1 in Tris–HCl, pH 7.0 buffer is shown (Figure 2A). The negative change in heat-flow shows the reaction is exothermic. The largest displacement of the baseline (120–160 s, Figure 2A) corresponds to the maximum catalytic rate under enzyme saturation conditions, while the decay portion of the curve (160–300 s) reflects gradual depletion of the substrate until fully consumed. The apparent enthalpy change (Δ*H*_app_) of each reaction was calculated by integrating the area under the entire titration curve and normalised for substrate consumed (in moles). The Δ*H*_app_ values for NDM-1 catalysed hydrolysis of penicillin G, cefazolin and imipenem at pH 7.0, 25°C were determined to be –27.9, –28.3 and –32.5 kcal/mol, respectively (Table 1). The measured heat reflects the total enthalpy of all events in the reaction vessel, including both the intrinsic enthalpy change of the reaction and the ionisation enthalpy of the buffer used. To determine the Δ*H*_int_ of each antibiotic, the reaction was performed in buffers having different ionisation enthalpies at pH 7.0. By plotting Δ*H*_app_ as a function of Δ*H*_ion_ specific to each buffer used (Figure 2B), it is apparent that there is a decrease in Δ*H*_app_ with the increase of Δ*H*_ion_, indicating NDM-1 catalysed hydrolysis of penicillin G involves exchanging of proton(s) with the buffer solution. The same result was obtained for cefazolin and imipenem. The number of exchanging protons (*n*) was determined as the slope of the fitted plots, −0.87 (± 0.10) for penicillin G and −1.24 (± 0.02) for imipenem, in agreement with previous data on metallo-β-lactamase L1 that approximately one proton is released to buffer during catalysis with the divergence from unity depending on the p*K*_a_ of the amino group in the hydrolysis product [36]. However, the hydrolysis of cefazolin by NDM-1 apparently involves a different mechanism, with two protons (*n* = −1.89 ± 0.04) being taken up by the buffer. The intrinsic enthalpy (Δ*H*_int_) of each antibiotic was determined as the *Y*-axis intercept, and values were −18.3 ± 0.73 kcal/mol for penicillin G, −18.4 ± 0.12 kcal/mol for imipenem, and a significantly lower value of −5.20 ± 0.04 kcal/mol for cefazolin. Based on existing data, the hydrolyses of these β-lactams by NDM-1 involve different processes [45–47]. Since NDM-1 catalysed hydrolysis of penicillin G and imipenem at pH 7.0 are accompanied by a similar intrinsic enthalpy change and both are associated with the release of one proton, it is reasonable to speculate that these two compounds use the same mechanism and the proton released is likely derived from ionisation of the new carboxylic acid generated at C-7 after β-lactam ring opening (Figure 3A,B). In comparison, a significant smaller intrinsic enthalpy change and one additional proton exchanged during cefazolin hydrolysis indicates a different pathway is adopted by NDM-1 in the hydrolysis of this compound. Indeed, it is known that for some cephalosporins, following the opening of the β-lactam ring, the anionic nitrogen intermediate is expected to undergo β-elimination of the leaving group with fragmentation of the initial product [45–48]. Hence, it is possible that the intrinsic enthalpy change of cefazolin turnover also involves heat change in this subsequent elimination reaction and the whole process generates one proton from ionisation of the new carboxylic acid at C-8 and a second proton from ionisation of the 2-thiothiadizole moiety generated by a β-elimination of that group from the C-3 side chain (Figure 3C).

**Table 1:** Thermodynamic and kinetic data of the hydrolysis of three β-lactam antibiotics catalysed by NDM-1 derived using ITC.

Compound	Δ*H*_app_ (kcal/mol)	*n*	Δ*H*_int_ (kcal/mol)	*k*_cat_ (s^−1^)	*K*_M_ (μM)	*k*_cat_/*K*_m_ (s^−1^ μM^−1^)	*k*_cat_/*K*_M_-UV^1^ (s^−1^ μM^−1^)
**Penicillin G**	−27.9 ± 0.01	−0.87 ± 0.10	−18.3 ± 0.73	54 ± 1	139 ± 12	0.39	0.68
**Cefazolin**	−28.3 ± 0.97	−1.89 ± 0.04	−5.20 ± 0.04	42 ± 1	237 ± 14	0.18	−
**Imipenem**	−32.5 ± 0.08	−1.24 ± 0.02	−18.4 ± 0.12	23 ± 1	168 ± 28	0.14	0.21

1 From Yong et al. [15] using the traditional UV-spectroscopic assay. Reactions were performed at 30°C in buffer 10 mM HEPES, pH 7.5.

**Figure 4:**
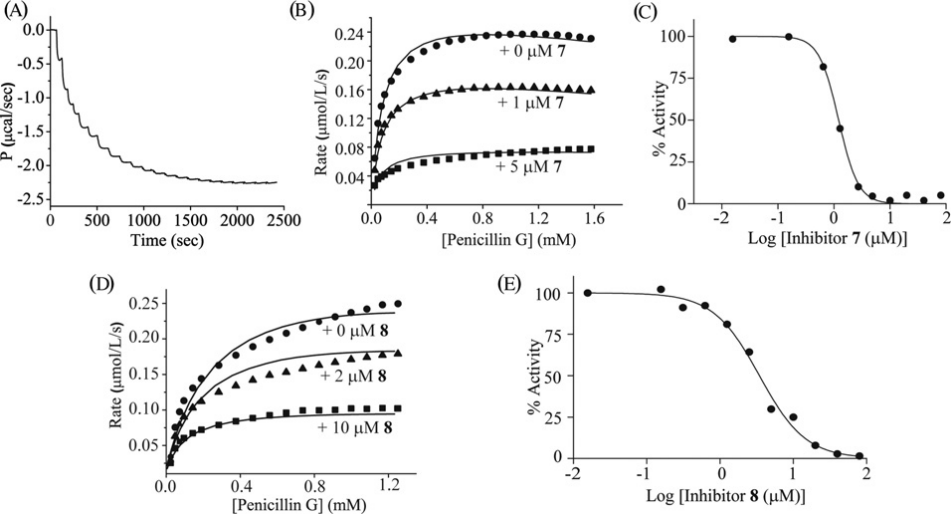
Enzyme kinetics and inhibition of NDM-1 catalysed penicillin G hydrolysis using a multiple injection ITC assay and a UV-spectroscopic assay (**A**) Raw heat change observed in the calorimetric assay, obtained by titrating 10 mM penicillin G into 10 nM NDM-1 with 23 injections. (**B** and **D**) Global fitting of NDM-1 enzymatic activity in the absence (circles) and presence (triangles and squares) of compounds **7** and **8** as an enzyme inhibitor using data from the multiple injection ITC assay. Baseline displacement of the thermal power in Figure 4(A) was converted into the reaction rate and plotted according to the Michaelis–Menten equation to obtain values of *K*_M_, *k*_cat_, *K*_ic_ and *K*_iu_. (**C** and **E**) IC_50_ of compounds **7** and **8** determined using a UV-spectroscopic method with inhibitor concentrations from 16 nM to 40 μM.

The calorimetric data for the titration of NDM-1 with penicillin G following a multiple injection method to obtain kinetic data is shown (Figure 4A). The process involves 23 successive injections of antibiotic solution into NDM-1 solution. A time interval of 120 s allows the heat flow to reach a brief plateau after each injection to mimic steady-state conditions of reaction (the enzyme is fully saturated and <5% of the substrate is depleted). Each injection of the substrate solution further increases the magnitude of heat change offset. The rate of heat change (d*Q*/d*t*) at each substrate concentration was determined as the displacement between the original baseline before the first injection and the new baseline after each injection. This value was then converted into enzyme turnover rates and fitted to the Michaelis–Menten equation to obtain the parameters *K*_M_ and *k*_cat_ (Figure 4B). As shown in Table 1, NDM-1 has a higher preference for penicillin G (*K*_M_ = 139 μM) as substrate, followed by imipenem (*K*_M_ = 168 μM) and cefazolin (*K*_M_ = 237 μM), and the overall catalytic efficiency of NDM-1 towards penicillin G (*k*_cat_/*K*_M_ = 0.39 s^−1^ μM^−1^) is also 2-fold higher than that of the cefazolin (*k*_cat_/*K*_M_ = 0.18 s^−1^ μM^−1^) and almost 3-fold higher than that of imipenem (*k*_cat_/*K*_M_ = 0.14 s^−1^ μM^−1^), at pH 7.0, 25°C. These results agree with previous data obtained in a UV-spectroscopic assay where the *k*_cat_/*K*_M_ for penicillin G was determined as 0.68 s^−1^ μM^−1^, and the value for imipenem was determined as 0.21 s^−1^ μM^−1^, measured in 10 mM HEPES, pH 7.5 buffer at 30°C [15].

After the calorimetric assay for steady-state kinetic study of NDM-1 was established, experiments were performed by pre-incubating different concentrations of inhibitor with NDM-1 for 30 min prior to substrate titration. As shown (Figure 4B), there is a progressive decrease in enzyme turnover rates with increase in concentration of **7** from 0 to 5 μM. Global fitting of the data to the general inhibition equation gives a competitive inhibition constant *K*_ic_ of 2.17 ± 0.48 μM and a similar uncompetitive inhibition constant *K*_iu_ of 2.07 ± 0.06 μM, showing **7** is a potent mixed inhibitor of NDM-1. The IC_50_ value of **7**, determined using a conventional UV-spectroscopic method, compares well with our inhibition data from ITC experiments (Table 2), with an IC_50_ value of 1.13 ± 0.04 μM for this compound. Our results, in conjunction with a previous report that the presence of 100 μM of this compound was capable of reducing enzyme activity of a different class B1 IMP-1 towards imipenem up to 2%, with barely any effect on BcII, VIM-2, or VIM-4 from the same class of MβLs [37], highlights this compound as having a good degree of selectivity for enzymes within class B1 MβLs. By comparison, **8** showed a much weaker competitive inhibition constant of *K*_ic_ = 152 ± 6 μM and a slightly larger uncompetitive inhibition constant of *K*_iu_ = 5 ± 0.46 μM (Figure 4C and Table 2), indicating the mechanism of **8** inhibition at pH 7.0 is predominantly uncompetitive, and the inhibitor binds to the enzyme–substrate complex instead of to the free enzyme. This *K*_iu_ is in good accord with the IC_50_ of 3.45 ± 0.26 μM for NDM-1 (Figure 4E), and also in agreement with previous data that this compound is a broad-spectrum inhibitor of MβLs with strong inhibitory potency towards class B1 CcrA (*K*_i_ = 0.64 μM), class B2 ImiS (*K*_i_ = 7.1 μM) and class B3 L1 (*K*_i_ = 1.8 μM) [39]. Of the dicarboxylic acid compounds tested, **5** and **6** have the weakest inhibitory activity towards NDM-1, in a mainly competitive mode, with *K*_ic_ = 365 ± 72 μM and IC_50_ = 409 ± 16 μM for **5** and *K*_ic_ = 198 ± 50 μM and IC_50_ = 232 ± 5 μM for **6**. In addition, as a control, the *K*_ic_ of **1** was determined to be 1.36 ± 0.79 μM by ITC and the UV-spectroscopic IC_50_ was 2.38 ± 0.64 μM, which are consistent with previously reported values of *K*_i_ = 1.3 μM and IC_50_ = 7.9 μM [24,25]. Taken together, the overall ranking of the tested inhibitors agrees well in both the ITC assay and the UV-spectroscopic assay, with **1, 7** and **8** being the most potent single-digit micro-molar inhibitors of NDM-1, while **5** and **6** were significantly weaker inhibitors by almost two orders of magnitude.

**Table 2:** Inhibition of NDM-1 by carboxylic acid compounds

Inhibitor	IC_50_ (μM)^1^	*K*_ic_ (μM)^2^	*K*_iu_ (μM)^2^
**1**	2.38 ± 0.64	1.36 ± 0.79	178 ± 21
**5**	409 ± 16	365 ± 72	993 ± 107
**6**	232 ± 5	198 ± 50	638 ± 94
**7**	1.13 ± 0.04	2.17 ± 0.48	2.07 ± 0.06
**8**	3.45 ± 0.26	152 ± 6	5 ± 0.46

1 Data were from the UV-spectroscopic assay in the present study. Experiments were performed at 25°C in buffer 50 mM Tris–HCl, pH 7.0, 100 mM NaCl.

2 Data were from the ITC inhibition assay in the present study. Experiments were performed at 25°C in buffer 50 mM Tris–HCl, pH 7.0, 100 mM NaCl.

**Figure 5:**
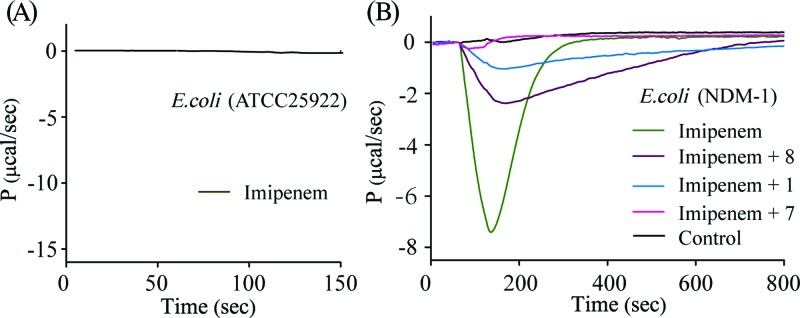
Heat flow data for the inhibition of NDM-1 in a cell-based calorimetric assay (**A**) Raw calorimetric trace for titrating imipenem (20 μl 1 mM) into suspensions of a reference *E. coli* (ATCC25922, no β-lactamase production) at OD = 4 in Tris–HCl buffer. (**B**) Overlaid calorimetric traces of titrating imipenem (20 μl 1 mM) into suspensions of an NDM-1-producing clinical *E. coli* strain at OD = 4 in the absence and presence of 400 μM inhibitors **1, 7** and **8**. Control experiment was performed by injecting buffer into bacterial suspensions.

A cell-based ITC assay was further performed with the more potent compounds **7** and **8**, using **1** as a reference, to ascertain whether these inhibitors can retard NDM-1 hydrolysis of imipenem in a cellular environment using live bacteria, in a manner similar to the reported UV–vis approach [49]. The raw data of titration of cell suspensions of a carbapenemase-negative strain (ATCC 25922) with imipenem (Figure 5A) reveal negligible heat change. In comparison, titration of imipenem into cell suspensions of an NDM-1–positive clinical *E. coli* strain (*E. coli*–NDM-1) led to a considerable negative change of heat-flow, much like the calorimetric curves with purified NDM-1 (Figure 5B). This strongly suggests that the derived heat-change in the calorimetric assay with *E. coli*–NDM-1 arises from enzyme-catalysed hydrolysis of imipenem. Cell-based inhibition assays were then performed by titrating imipenem with *E. coli*–NDM-1 in the presence of 400 μM of compounds **1, 7** and **8** (Figure 5B). All tested compounds were capable of inhibiting the turnover rate of imipenem and postponing the time required for complete consumption of the substrate, with an efficacy in the order of **7** > **1** > **8**. The MICs of imipenem on this clinical *E. coli*–NDM-1 isolate, in the absence and presence of compounds **1, 7** and **8**, were next determined (Table 3). The addition of 200–400 μM inhibitors alone showed no effect on bacterial growth, indicating these compounds have no antimicrobial activity at concentrations tested. However, when **7** and **8** were administered in combination with imipenem, the resistant strain was re-sensitised towards imipenem. The presence of 400 μM of **7** was able to restore the susceptibility of *E. coli*–NDM-1 towards imipenem, by lowering the MICs of imipenem from 16 to 1 μg/ml. Compound **8** at 400 μM reduced the MICs by 2-fold and shows a weaker synergy with imipenem. Surprisingly, reference compound **1** failed to show any synergistic effect with imipenem at the tested concentrations, notwithstanding its superior inhibitory potency in the above *in vitro* experiments**.** The possibility of **1** inactivation due to slow oxidation of –SH during overnight incubation with bacterial suspensions needs to be investigated.

**Table 3:** MICs of a clinical *E. coli*–NDM-1 strain towards imipenem and imipenem-inhibitor combination

	MICs (μg/ml) of imipenem in combination with **1, 7** and **8** at various concentrations		
	0 μM	200 μM	400 μM
*E.coli*–NDM-1	16		
*E.coli*–NDM-1 + **1**		16	16
*E.coli*–NDM-1 + **7**		4	1
*E.coli*–NDM-1 + **8**		16	8

## Conclusion

NDM-1 is a class B1 metallo-β-lactamase commonly found in clinical multi-drug resistant pathogens that catalyse the hydrolysis of β-lactam antibiotics, leading to inactivation of these bactericides. We have used a new ITC approach to study the thermodynamic data and hydrolytic activity of NDM-1 towards individual β-lactams from three families, penicillin G, cefazolin and imipenem, coupled to the inhibitory potency of four low-molecular-weight dicarboxylic acid compounds to promote their activity. Our results reveal that NDM-1 catalysed hydrolysis of penicillin G, cefazolin and imipenem follow different mechanisms, with cefazolin showing a greatly reduced intrinsic enthalpy change as well as release of a second proton during β-lactamase turnover. Both ITC and UV-spectroscopic experiments identify pyridine-2,6-dicarboxylic acid and thiazolidine-2,4-dicarboxylic acid as good inhibitors of NDM-1. These compounds also show good potency in inhibiting NDM-1 hydrolysis of imipenem at the cellular level and re-sensitising a clinical strain of *E. coli*–NDM-1 towards imipenem, suggesting they are promising leads for future development of more potent inhibitors of NDM-1. The possibility that the activity of pyridine-2,6-dicarboxylic acid and thiazolidine-2,4-dicarboxylic acid results from zinc coordination is clearly reminiscent of the outstanding development of ACE inhibitors such as Captopril from the work of Ondetti and Kushman and merits active development [50], while recent crystallographic analysis has identified the sulphur atom of Captopril bridging the two zinc atoms in SMB-1 (PDB: 5aya), and bidentate coordination to a single zinc atom involving sulphur has been exemplified for blaNDM-1 (PDB: 5a5z) [51], and underpins current work based on cysteine for inhibitor development [52]. The well-known power of pyridinecarboxylic acids to zinc has been exemplified by picolinic acid for ACC oxidase [53], but there is no structural report of enzymatic zinc chelation by compound **7**. It is thus reasonable to propose that the inhibitory effect of compounds **7** and **8** may result from a combination of monodentate and bidentate coordination to the two zincs in NDM-1 and merits detailed structural analysis. Compared with D-(−)-tartaric acid and fumaric acid, the superior inhibitory potency of pyridine-2,6-dicarboxylic acid and thiazolidine-2,4-dicarboxylic acid with NDM-1 are likely ascribed to two aspects. Firstly, constrains imposed by the pyridine and thiazolidine rings may help to arrange the attached carboxylate groups for more favourable hydrogen-bonding/ionic interactions with enzyme active site residues. Secondly, the pyridine and thiazolidine rings may also directly contribute to stronger binding by making hydrophobic interactions with nonpolar residues at NDM-1 active site. In the perspective of generating broad-spectrum inhibitors of MβLs for combination therapy, the mechanistic complexity of antibiotic hydrolysis and different modes of inhibitor binding, as highlighted in this work with NDM-1, pose a huge challenge. This work also endorses the ITC assay as a reliable method to obtain kinetic data on enzyme catalysis and inhibition at both protein and cellular levels, with the additional advantage of providing important thermodynamic information on enzyme catalysis. The ITC assay presented here is expected to be adopted as a general method to facilitate mechanistic studies of other clinical β-lactamases and the evaluation of potential inhibitors.

## Abbreviations

CLSI: Clinical and Laboratory Standards Institutes
ESBLs: extended-spectrum β-lactamases
ITC: isothermal titration calorimetry
MIC: minimum inhibitory concentration
MβLs: metallo-β-lactamases
NDM-1: New Delhi metallo-β-lactamase
PBPs: penicillin binding proteins

## References

[1] Fisher J.F., Meroueh S.O. and Mobashery S. (2005) Bacterial resistance to β-lactam antibiotics: compelling opportunism, compelling opportunity. Chem. Rev. 105, 395–424 10.1021/cr030102i 15700950

[2] Papp-Wallace K.M., Endimiani A., Taracila M.A. and Bonomo R.A. (2011) Carbapenems: past, present, and future. Antimicrob. Agents Chemother. 55, 4943–4960 10.1128/AAC.00296-11 21859938 PMC3195018

[3] Harris P.N.A., Tambyah P.A. and Paterson D.L. (2015) β-Lactam and β-lactamase inhibitor combinations in the treatment of extended-spectrum β-lactamase producing *Enterobacteriaceae*: time for a reappraisal in the era of few antibiotic options? Lancet Infect. Dis. 15, 475–485 10.1016/S1473-3099(14)70950-8 25716293

[4] Nordmann P. and Poirel L. (2002) Emerging carbapenemases in Gram-negative aerobes. Clin. Microbiol. Infect. 8, 321–331 10.1046/j.1469-0691.2002.00401.x 12084099

[5] Delcour A.H. (2009) Outer membrane permeability and antibiotic resistance. Biochim. Biophys. Acta 1794, 808–816 10.1016/j.bbapap.2008.11.005 19100346 PMC2696358

[6] Webber M.A. (2002) The importance of efflux pumps in bacterial antibiotic resistance. J. Antimicrob. Chemother. 51, 9–11 10.1093/jac/dkg050 12493781

[7] Gehrlein M., Leying H., Cullmann W., Wendt S. and Opferkuch W. (1991) Imipenem resistance in *Acinetobacter baumanii* is due to altered penicillin-binding proteins. Chemotherapy 37, 405–412 10.1159/000238887 1760939

[8] King D., Sobhanifar S., Strynadka N. (2017) The mechanisms of resistance to β-lactam antibiotics. In Handbook Of Antimicrobial Resistance, , 177–201, Springer, New York.

[9] Bonomo R.A., Burd E.M., Conly J., Limbago B.M., Poirel L. and Segre J.A. (2017) Carbapenemase-producing organisms: a global scourge!. Clin. Infect. Dis. 10.1093/cid/cix893 PMC5884739 29165604

[10] Queenan A.M. and Bush K. (2007) Carbapenemases: the versatile β-lactamases. Clin. Microbiol. Rev. 20, 440–458 10.1128/CMR.00001-07 17630334 PMC1932750

[11] Docquier J.D. and Mangani S. (2017) An update on β-lactamase inhibitor discovery and development. Drug Resist. Update 70, 651–679 10.1016/j.drup.2017.11.002 29499835

[12] Ehmann D.E., Jahic H., Ross P.L., Gu R.F., Hu J. and Durand-Reville T.F. (2013) Kinetics of avibactam inhibition against class A, C, and D β-lactamases. J. Biol. Chem. 288, 27960–27971 10.1074/jbc.M113.485979 23913691 PMC3784710

[13] Sharma R., Park T.E. and Moy S. (2013) Ceftazidime-avibactam: a novel cephalosporin/β-lactamase inhibitor combination. Drugs 73, 159–177 10.1007/s40265-013-0013-7 23371303

[14] Hirsch E.B., Ledesma K.R., Chang K.T., Schwartz M.S., Motyl M.R. and Tam V.H. (2012) In vitro activity of MK-7655, a novel β-lactamase inhibitor, in combination with imipenem against carbapenem-resistant gram-negative bacteria. J. Antimicrob. Chemother. 56, 3753–3757 10.1128/AAC.05927-11 PMC3393460 22526311

[15] Yong D., Toleman M.A., Giske C.G., Cho H.S., Sundman K. and Lee K. (2009) Characterization of a new metallo- β-lactamase gene, bla(NDM-1), and a novel erythromycin esterase gene carried on a unique genetic structure in *Klebsiella pneumoniae* sequence type 14 from India. Antimicrob. Agents Chemother. 53, 5046–5054 10.1128/AAC.00774-09 19770275 PMC2786356

[16] Khan A.U., Maryam L. and Zarrilli R. (2017) Structure, genetics and worldwide spread of New Delhi metallo-β-lactamase (NDM): a threat to public health. BMC Microbiol. 17, 101 10.1186/s12866-017-1012-8 28449650 PMC5408368

[17] Johnson A.P. and Woodford N. (2013) Global spread of antibiotic resistance: the example of New Delhi metallo-β-lactamase (NDM)-mediated carbapenem resistance. J. Med. Microbiol. 62, 499–513 10.1099/jmm.0.052555-0 23329317

[18] Lisa M.N., Palacios A.R., Aitha M., Gonzalez M.M., Moreno D.M., Crowder M.W. (2017) A general reaction mechanism for carbapenem hydrolysis by mononuclear and binuclear metallo-β-lactamases. Nat. Commun. 8, 538 10.1038/s41467-017-00601-9 28912448 PMC5599593

[19] Yang H., Aitha M., Marts A.R., Hetrick A., Bennett B. and Crowder M.W. (2014) Spectroscopic and mechanistic studies of heterodimetallic forms of metallo-β-lactamase NDM-1. J. Am. Chem. Soc. 136, 7273–7285 10.1021/ja410376s 24754678 PMC4046764

[20] Berrazeg M., Diene S.M., Medjahed L., Parola P., Drissi M. and Raoult D. (2014) New Delhi metallo-β-lactamase around the world: an eReview using Google maps. Eurosurveillance 19, 2–15 10.2807/1560-7917.ES2014.19.20.20809 24871756

[21] Brem J., Cain R., Cahill S., McDonough M.A., Clifton I.J., Jimenez-Castellanos J.C. (2016) Structural basis of metallo-β-lactamase, serine-β-lactamase and penicillin-binding protein inhibition by cyclic boronates. Nat. Commun. 7, 12406 10.1038/ncomms12406 27499424 PMC4979060

[22] Fast W. and Sutton L.D. (2013) Metallo-β-lactamase: inhibitors and reporter substrates. Biochim. Biophys. Acta 1834, 1648–1659 10.1016/j.bbapap.2013.04.024 23632317

[23] Groundwater P.W., Xu S., Lai F., Varadi L., Tan J. and Perry J.D. (2016) New Delhi metallo-β-lactamase-1: structure, inhibitors and detection of producers. Future Med. Chem. 8, 993–1012 10.4155/fmc-2016-0015 27253479

[24] Guo Y., Wang J., Niu G., Shui W., Sun Y., Zhou H. (2011) A structural view of the antibiotic degradation enzyme NDM-1 from a superbug. Protein Cell 2, 384–394 10.1007/s13238-011-1055-9 21637961 PMC4875342

[25] King D.T., Worrall L.J., Gruninger R. and Strynadka N.C. (2012) New Delhi metallo-β-lactamase: structural insights into β-lactam recognition and inhibition. J. Am. Chem. Soc. 134, 11362–11365 10.1021/ja303579d 22713171

[26] Sahoo J. and Paidesetty S.K. (2015) Antimicrobial, analgesic, antioxidant and in silico study of synthesized salicylic acid congeners and their structural interpretation. Egypt J. Basic Appl. Sci. 2, 268–280 10.1016/j.ejbas.2015.07.006

[27] Livermore D.M., Mushtaq S., Morinaka A., Ida T., Maebashi K. and Hope R. (2013) Activity of carbapenems with ME1071 (disodium 2,3-diethylmaleate) against *Enterobacteriaceae* and *Acinetobacter* spp. with carbapenemases, including NDM enzymes. J. Antimicrob. Chemother. 68, 153–158 10.1093/jac/dks350 22945917

[28] von Nussbaum F. and Schiffer G. (2014) Aspergillomarasmine A, an inhibitor of bacterial metallo-β-lactamases conferring blaNDM and blaVIM resistance. Angew. Chem., Int. Ed. Engl. 53, 11696–11698 10.1002/anie.201407921 25256630

[29] King A.M., Reid-Yu S.A., Wang W., King D.T., De Pascale G., Strynadka N.C. (2014) Aspergillomarasmine A overcomes metallo-β-lactamase antibiotic resistance. Nature 510, 503–506 10.1038/nature13445 24965651 PMC4981499

[30] Todd M.J. and Gomez J. (2001) Enzyme kinetics determined using calorimetry: a general assay for enzyme activity? Anal. Biochem. 296, 179–187 10.1006/abio.2001.5218 11554713

[31] Freyer M.W. and Lewis E.A. (2008) Isothermal titration calorimetry: experimental design, data analysis, and probing macromolecule/ligand binding and kinetic interactions. Methods Cell. Biol. 84, 79–113 10.1016/S0091-679X(07)84004-0 17964929

[32] Morin P.E. and Freire E. (1991) Direct calorimetric analysis of the enzymatic activity of yeast cytochrome *c* oxidase. Biochemistry 30, 8494–8500 10.1021/bi00098a030 1653014

[33] Frasca V. (2016) Using isothermal titration calorimetry techniques to quantify enzyme kinetics. Ind. Biotechnol. 12, 207–211 10.1089/ind.2016.29040.vfr

[34] Hansen L.D., Transtrum M.K., Quinn C. and Demarse N. (2016) Enzyme-catalyzed and binding reaction kinetics determined by titration calorimetry. Biochim. Biophys. Acta 1860, 957–966 10.1016/j.bbagen.2015.12.018 26721335

[35] Mazzei L., Ciurli S. and Zambelli B. (2016) Isothermal titration calorimetry to characterize enzymatic reactions. Methods Enzymol. 215–236 10.1016/bs.mie.2015.07.022 26794356

[36] Wang W.J., Wang Q., Zhang Y., Lu R., Zhang Y.L. and Yang K.W. (2017) Characterization of β-lactamase activity using isothermal titration calorimetry. Biochim. Biophys. Acta 1861, 2031–2038 10.1016/j.bbagen.2017.04.011 28454737

[37] Horsfall L.E., Garau G., Lienard B.M., Dideberg O., Schofield C.J., Frere J.M. (2007) Competitive inhibitors of the CphA metallo-β-lactamase from *Aeromonas hydrophila*. Antimicrob. Agents. Chemother 51, 2136–2142 10.1128/AAC.00866-06 17307979 PMC1891371

[38] Chen A.Y., Thomas P.W., Stewart A.C., Bergstrom A., Cheng Z. and Miller C. (2017) Dipicolinic acid derivatives as inhibitors of New Delhi metallo-β-lactamase-1. J. Med. Chem. 60, 7267–7283 10.1021/acs.jmedchem.7b00407 28809565 PMC5599375

[39] Feng L., Yang K.W., Zhou L.S., Xiao J.M., Yang X., Zhai L. (2012) N-heterocyclic dicarboxylic acids: broad-spectrum inhibitors of metallo-β-lactamases with co-antibacterial effect against antibiotic-resistant bacteria. Bioorg. Med. Chem. Lett. 22, 5185–5189 10.1016/j.bmcl.2012.06.074 22796180

[40] Payne D.J., Bateson J.H., Gasson B.C., Proctor D., Khushi T. and Farmer T.H. (1997) Inhibition of metallo-β-lactamases by a series of mercaptoacetic acid thiol ester derivatives. Antimicrob. Agents Chemother. 41, 135–140 8980769 10.1128/aac.41.1.135 PMC163674

[41] Yang H., Aitha M., Hetrick A.M., Richmond T.K., Tierney D.L. and Crowder M.W. (2012) Mechanistic and spectroscopic studies of metallo-β-lactamase NDM-1. Biochemistry 51, 3839–3847 10.1021/bi300056y 22482529

[42] Zhang Y.L., Yang K.W., Zhou Y.J., LaCuran A.E., Oelschlaeger P. and Crowder M.W. (2014) Diaryl-substituted azolylthioacetamides: inhibitor discovery of New Delhi metallo-β-lactamase-1 (NDM-1). Chem. Med. Chem. 9, 2445–2448 10.1002/cmdc.201402249 25048031

[43] Fukada H. and Takahashi K. (1998) Enthalpy and heat capacity changes for the proton dissociation of various buffer components in 0.1 M potassium chloride. Proteins 33, 159–166 10.1002/(SICI)1097-0134(19981101)33:2%3c159::AID-PROT2%3e3.0.CO;2-E 9779785

[44] CLSI. (2017) Performance Standards for Antimicrobial Susceptibility Testing: 27th Informational Supplement, Clinical Laboratory Standards Institute, Wayne, PA

[45] Vilanova B., Frau J., Donoso J., Munoz F. and Blanco F.G. (1997) β-Lactamase-catalysed hydrolysis of cephalexin: evolution of the cephalosporoate intermediate. J. Chem. Soc. 11, 2439–2444

[46] Strynadka N., Adachi H., Jensen S., Johns K., Sielecki A. and Betzel C. (1992) Molecular structure of the acyl-enzyme intermediate in β-lactam hydrolysis at 1.7 Å resolution. Nature 359, 700–705 10.1038/359700a0 1436034

[47] Feng H., Ding J., Zhu D., Liu X., Xu X. and Zhang Y. (2014) Structural and mechanistic insights into NDM-1 catalyzed hydrolysis of cephalosporins. J. Am. Chem. Soc. 136, 14694–14697 10.1021/ja508388e 25268575

[48] Pratt R.F. and Faraci W.S. (1986) Direct observation by proton NMR of cephalosporoate intermediates in aqueous solution during the hydrazinolysis and β-lactamase-catalyzed hydrolysis of cephalosporins with 3′-leaving groups: kinetics and equilibria of the 3′-elimination reaction. J. Am. Chem. Soc. 108, 5329–5333 10.1021/ja00277a044

[49] Yang K., Zhou Y., Ge Y. and Zhang Y. (2017) Real-time activity monitoring of New Delhi metallo-β-lactamase-1 in living bacterial cells by UV-Vis spectroscopy. Chem. Commun. 53, 8014–8017 10.1039/C7CC02774E 28664213

[50] Ondetti M., Rubin B. and Cushman D. (1977) Design of specific inhibitors of angiotensin-converting enzyme: new class of orally active antihypertensive agents. Science 196, 441–444 10.1126/science.191908 191908

[51] Klingler F.M., Wichelhaus T.A., Frank D., Cuesta-Bernal J., El-Delik J., Müller H.F. (2015) Approved drugs containing thiols as inhibitors of metallo-beta-lactamases: strategy to combat multidrug-resistant bacteria. J. Med. Chem. 58, 3626–3630 10.1021/jm501844d 25815530

[52] Bai C.G., Xu Y.T., Li N.N., Wang J.H., Yang C. and Chen Y. (2015) Cysteine and its derivatives as New Delhi metallo-β-lactamase-1 inhibitors. Curr. Enzyme Inhib. 11, 46–57 10.2174/1573408011666150408223245

[53] Sun X.Z., Li Y.R., He W.R., Ji C.G., Xia P.X. and Wang Y.C. (2017) Pyrazinamide and derivatives block ethylene biosynthesis by inhibiting ACC oxidase. Nat. Commun. 8, 15758 10.1038/ncomms15758 28604689 PMC5472784

